# Role of YAP and TAZ in pancreatic ductal adenocarcinoma and in stellate cells associated with cancer and chronic pancreatitis

**DOI:** 10.1038/srep16759

**Published:** 2015-11-16

**Authors:** Susan Morvaridi, Deepti Dhall, Mark I. Greene, Stephen J. Pandol, Qiang Wang

**Affiliations:** 1 Department of Medicine; Cedars-Sinai Medical Center, Los Angeles, CA 90048; 2 Pancreatic Research Program; Cedars-Sinai Medical Center, Los Angeles, CA 90048; 3 Department of Pathology and Laboratory Medicine; Cedars-Sinai Medical Center, Los Angeles, CA 90048; 4 Department of Pathology and Laboratory Medicine, Perelman School of Medicine, University of Pennsylvania, Philadelphia, PA 19104.

## Abstract

Pancreatic ductal adenocarcinoma (PDAC) is characterized by a fibrotic and inflammatory microenvironment that is formed primarily by activated, myofibroblast-like, stellate cells. Although the stellate cells are thought to contribute to tumorigenesis, metastasis and drug resistance of PDAC, the signaling events involved in activation of the stellate cells are not well defined. Functioning as transcription co-factors, Yes-associated protein (YAP) and its homolog transcriptional co-activator with PDZ-binding motif (TAZ) modulate the expression of genes involved in various aspects of cellular functions, such as proliferation and mobility. Using human tissues we show that YAP and TAZ expression is restricted to the centroacinar and ductal cells of normal pancreas, but is elevated in cancer cells. In particular, YAP and TAZ are expressed at high levels in the activated stellate cells of both chronic pancreatitis and PDAC patients as well as in the islets of Langerhans in chronic pancreatitis tissues. Of note, YAP is up regulated in both acinar and ductal cells following induction of acute and chronic pancreatitis in mice. These findings indicate that YAP and TAZ may play a critical role in modulating pancreatic tissue regeneration, neoplastic transformation, and stellate cell functions in both PDAC and pancreatitis.

Pancreatic cancer is the fourth leading cause of cancer-related death^1^,^2^,^3^. Pancreatic ductal adenocarcinoma (PDAC) comprises more than 85% of all pancreatic cancer and has extremely poor prognosis, with an overall five-year survival rate at less than 5%^2^,^4^. Chronic pancreatitis, a clinical syndrome of persistent pancreatic inflammation, is one of the leading risk factors for pancreatic cancer^5^,^6^. The normal exocrine pancreas primarily consists of acinar cells, which produce digestive enzymes, and ductal cells that form the lining of the secretory ductal system. Centroacinar cells are located at the junction between acinar cells and the terminal ductal epithelium. In addition, pancreatic stellate cells (PSCs) are myofibroblastlike cells that are normally quiescent but become activated in damaged pancreas and produce collagen, fibronectin and other fibrosis related proteins^7^,^8^. Both PDAC and chronic pancreatitis are characterized by a fibrotic and inflammatory microenvironment that is dominated by activated stellate cells.

The Hippo-YAP signaling pathway was initially identified as a mechanism involved in regulation of organ size and tissue growth and is more recently implicated in playing a role in cell proliferation, migration, stem cell self-renewal, and tissue regeneration^9^,^10^,^11^,^12^,^13^. In mammalian cells, YAP and its homolog TAZ (also known as WW Domain Containing Transcription Regulator 1, or WWTR1) function as transcriptional cofactors and the core of this signaling pathway^14^. The transcriptional activity of YAP and TAZ is subjected to negative regulation by a cascade of phosphorylation events, mediated by Mst1/2 and LATS1/2, leading to cytoplasmic sequestration or ubiquitin-mediated degradation^9^,^10^. In particular, YAP can be phosphorylated at S127 in a cell density-dependent manner and forms a more stable complex with the 14-3-3 proteins, thereby becoming retained in the cytoplasm^15^,^16^,^17^. YAP phosphorylation is mediated by signaling events are initiated from cell surface adhesion molecules, including E-cadherin-like molecules and proteins of the adherens junction and tight junction protein complexes^9^,^10^. In addition, YAP activity can be inhibited through the interactions with angiomotin (AMOT) family proteins, which lead to localization and sequestration of the YAP protein to tight junction^9^,^18^,^19^, or through interactions with PTPN14^10^,^20^,^21^,^22^,^23^, a non-receptor tyrosine phosphatase that is localized to the tight junction of epithelial cells^9^,^24^,^25^,^26^.

Here, we provide evidence that YAP and TAZ are present in normal pancreatic centroacinar and ductal cells, and are up regulated in pancreatic cancer cells and in the activated pancreatic stellate cells that define the stromal environment of chronic pancreatitis and pancreatic cancer. We also find significant increase in the cells of the islets of Langerhans in chronically inflamed but not normal pancreas. Moreover, YAP levels are increased in experimental acute and chronic pancreatitis. Our results support the notion that YAP and TAZ deregulation may play a role in pathogenesis of pancreatic diseases.

## Results

### YAP and TAZ/WWTR1 are primarily expressed in the centroacinar and ductal cells in normal human pancreas

**Figure 1:**
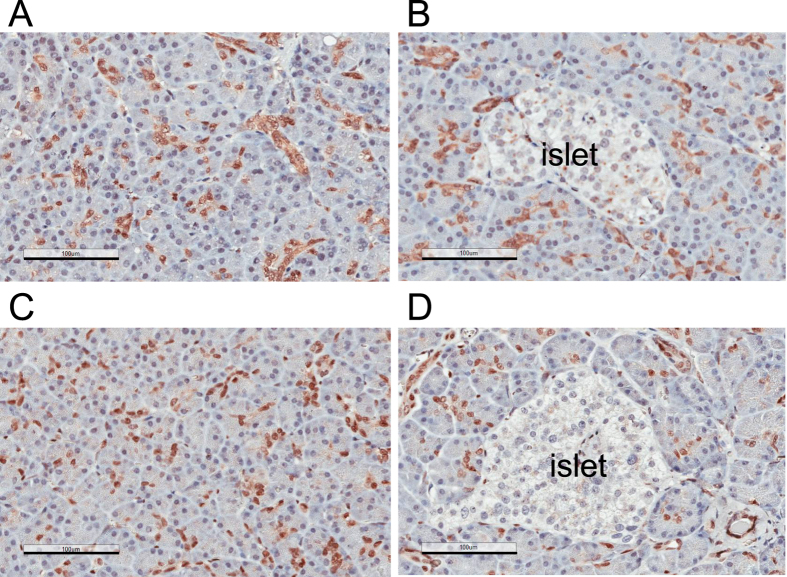
Immunohistochemistry analysis of YAP and TAZ/WWTR1 in normal human pancreas. (**A,B)** Immunohistochemistry analysis of normal human pancreatic tissue using anti-YAP antibody (H125); Two representative fields of the same sample are shown. Specimens obtained from an additional three individuals were also analyzed and shown in Supplemental Figure S2. The islet of Langerhans is indicated. Magnification: 20 x, error bar: 100 μm. **(C,D)** Immunohistochemistry analysis of normal human pancreatic tissue using anti-TAZ/WWTR1 antibody. Two representative fields of the same sample are shown. The islet of Langerhans is indicated. Magnification: 20 x, error bar: 100 μm.

We performed immunohistochemistry to examine the expression patterns of YAP and TAZ/WWTR1 in normal human pancreatic tissues obtained from four individuals. The YAP expression patterns resemble those of the centroacinar cells and ductal cells (Fig. 1), the subpopulations of the exocrine compartments that are implicated in having certain stem cell properties during tissue regeneration following injury^27^,^28^,^29^,^30^,^31^,^32^. In these cells, YAP and TAZ show diffused localization and the proteins can be found in both nuclei and cytoplasm (Fig. 1). In contrast, YAP is not expressed in acinar cells, or in islets of Langerhans (Fig. 1). These expression patterns of YAP were confirmed using three additional anti-YAP antibodies, including antibodies specific for the S127 phosphorylated form of YAP (Supplemental Figure S1). Furthermore, we observed similar centroacinar/ductal localization patterns for TAZ (Fig. 1).

**Figure 2:**
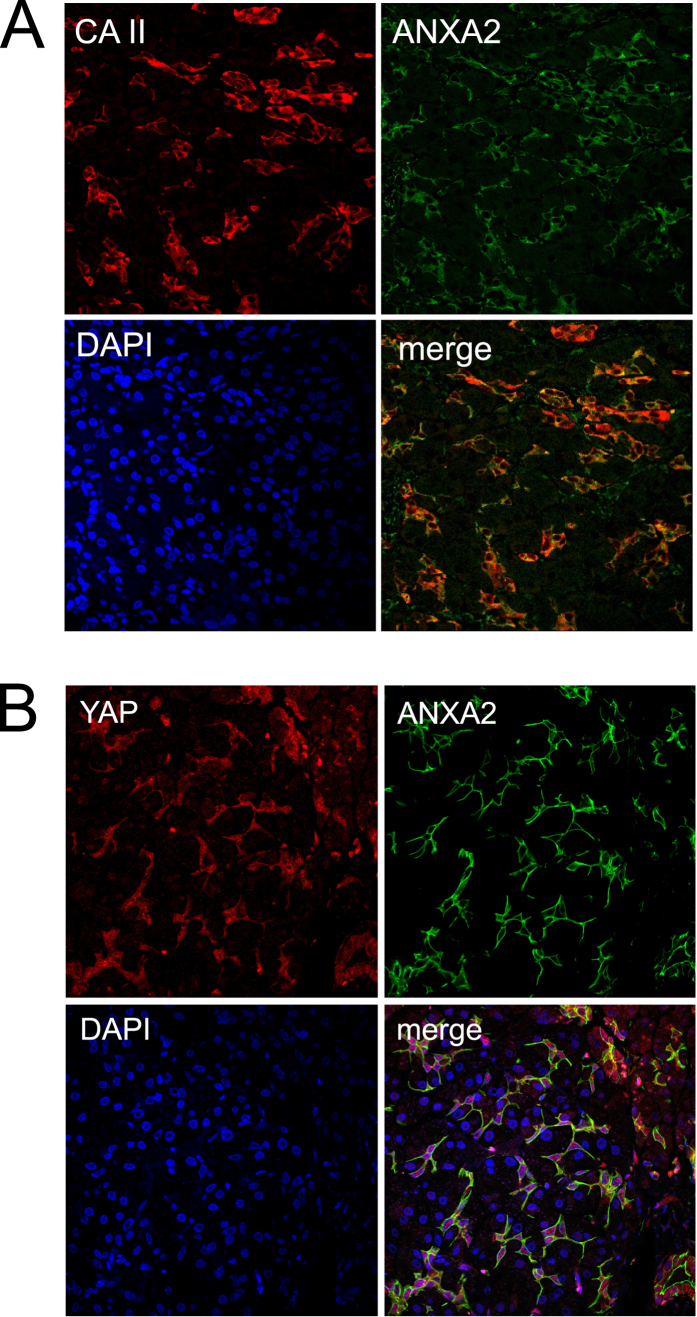
Immunofluorescence staining of YAP in normal human pancreas. (**A**) annexin A2 (ANXA2) (green), carbonic anhydrase II (CA II) (red), DAPI (blue); Magnification: 63 x. Note that ANXA2 is expressed in the same cells that are positive of CA II, a marker for pancreatic centroacinar and ductal cells. **(B)** YAP (anti-YAP, H-125) (red) and ANXA2 (green), DAPI (blue). Magnification: 63 x. Note that YAP and ANXA2 are co expressed in the same set of cells.

Immunofluorescence studies were carried out to determine whether YAP is co-expressed with established markers of centroacinar cells and ductal cells, such as carbonic anhydrase II (CA II)^33^,^34^,^35^. Similar to a previous study^36^, our study showed that annexin A2 (ANXA2) staining exhibits a pattern indicative of centroacinar and ductal cell specific expression (Supplemental Figure S4). In addition, we found that ANXA2 is co-expressed with CA II in normal pancreas and thus established it as another marker for the centroacinar and ductal cells (Fig. 2A and supplemental Table S1). Moreover, our results clearly show co-expression of YAP and ANXA2 in the normal pancreas (Fig. 2B and supplemental Table S1), which indicates that YAP expression is primarily restricted to the centroacinar and ductal cells. Notably, ANXA2 is predominantly localized to the plasma membrane in these cells, whereas YAP is primarily found in the cytoplasm and to a lesser extent in the nuclei (Fig. 2).

### YAP and TAZ expression in pancreatic intraepithelial neoplasia, PDAC and chronic pancreatitis

**Figure 3:**
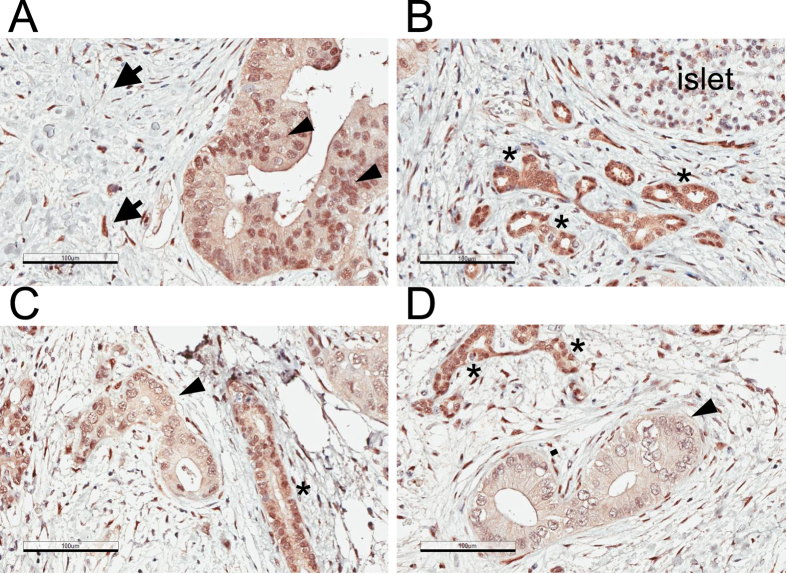
YAP expression in human pancreatic cancer tissues. (**A**–**D**) YAP staining (anti-YAP antibody, H125) in PDAC #1 tissue. Specimens obtained from an additional three PDAC individuals were also analyzed and shown in Supplemental Figure 2. Representative images of four different fields of the same sample are shown. Note that YAP can be detected in stellate cells (arrows), cancer cells (arrowheads) and in structures showing morphology of early PanIN or normal ducts (asterisks). Magnification: 20 x, error bar: 100 μm.

**Figure 4:**
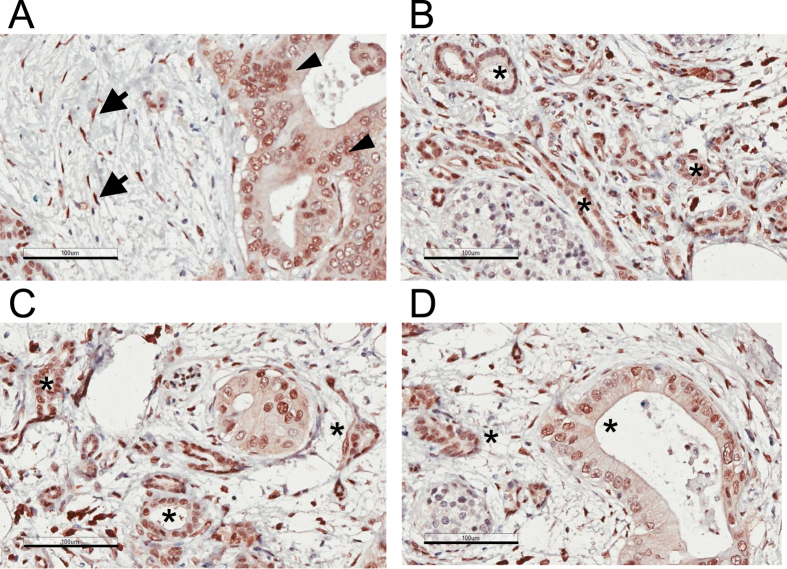
TAZ/WWTR1 expression in human pancreatic cancer tissues. (**A**–**D**) TAZ/WWTR1 in PDAC tissues (arrows: stellate cells; arrowheads: cancer cells; asterisks: PanIN or ducts with normal morphology). Representative images of four different fields of the same sample are shown. Magnification: 20 x, error bar: 100 μm.

Among the PDAC tissues collected from four individual patients, high levels of YAP were detected in pancreatic cancer cells that exhibit epithelial morphology (Fig. 3 and Supplemental Figure S2). Similar, the levels of TAZ are also elevated in PDAC (Fig. 4). In particular, YAP and TAZ can be detected in cells that show characteristics of pancreatic intraepithelial neoplasia (PanIN) (Figs 3 and 4), which represent the majority of the early neoplastic lesions^37^,^38^. Both YAP and TAZ show a more prominent nuclear localization pattern, which is consistent with the notion that these proteins are functionally activated. Moreover, a fraction of stromal cells are also stained positive for YAP and TAZ (Figs 3 and 4).

**Figure 5:**
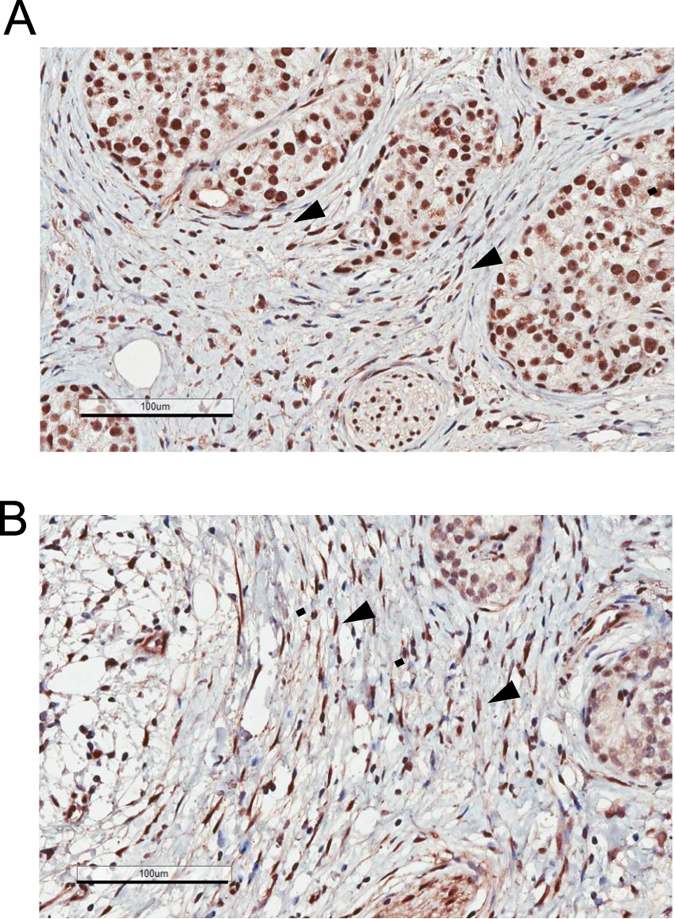
YAP and TAZ/WWTR1 expression in human chronic pancreatitis (CP) tissues. **(A)** Chronic pancreatitis tissue isolated from CP patient #1, stained using anti-YAP antibody (H125). Specimens obtained from an additional three individuals were also analyzed and shown in Supplemental Figure S3. Representative images are shown. **(B)** Chronic pancreatitis tissue (CP#1) stained using anti-WWTR1/TAZ antibody. The arrowheads indicate expression of YAP or TAZ in stellate cells. Magnification: 20 x, error bar: 100 μm.

Notably, in tissues of chronic pancreatitis, YAP and TAZ levels are expressed in a sub-population of stromal cells (Fig. 5 and Supplemental Figure S3). YAP and TAZ levels also appear to be elevated in the islets of Langerhans in the chronic pancreatitis tissue in contrast to normal pancreas. The localization is predominantly nuclear in the islet cells (Fig. 5 and Supplemental Figure S3).

### YAP is expressed at high levels in activated stellate cells associated with chronic pancreatitis and PDAC

**Figure 6:**
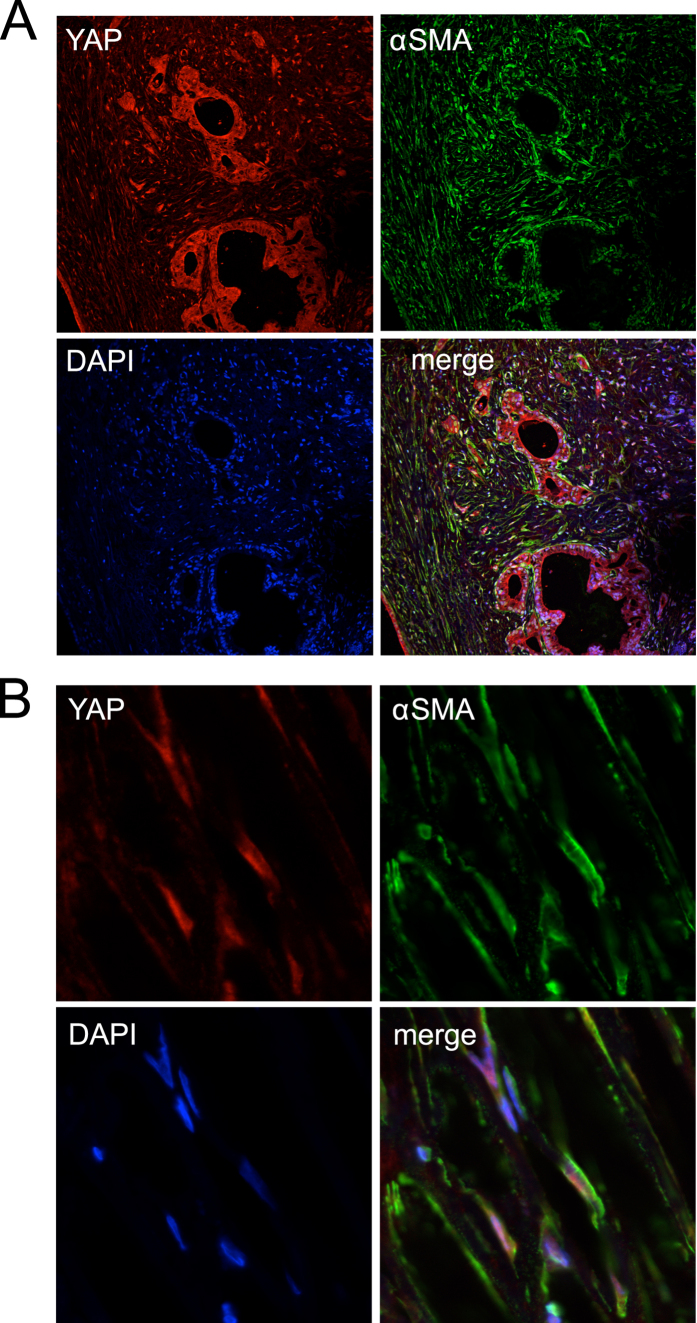
Immunofluorescence analysis of YAP in stellate cells of human PDAC tissues. (**A**) YAP (red) and α-SMA (green), and DAPI (blue). Note that YAP can be detected in both the cancer cells and the stromal cells. Magnification: 20 x. **(B)** Image of PDAC tissue stained in the same way as in (**A**), showing a field predominantly of stellate cells. Magnification: 63x.

**Figure 7:**
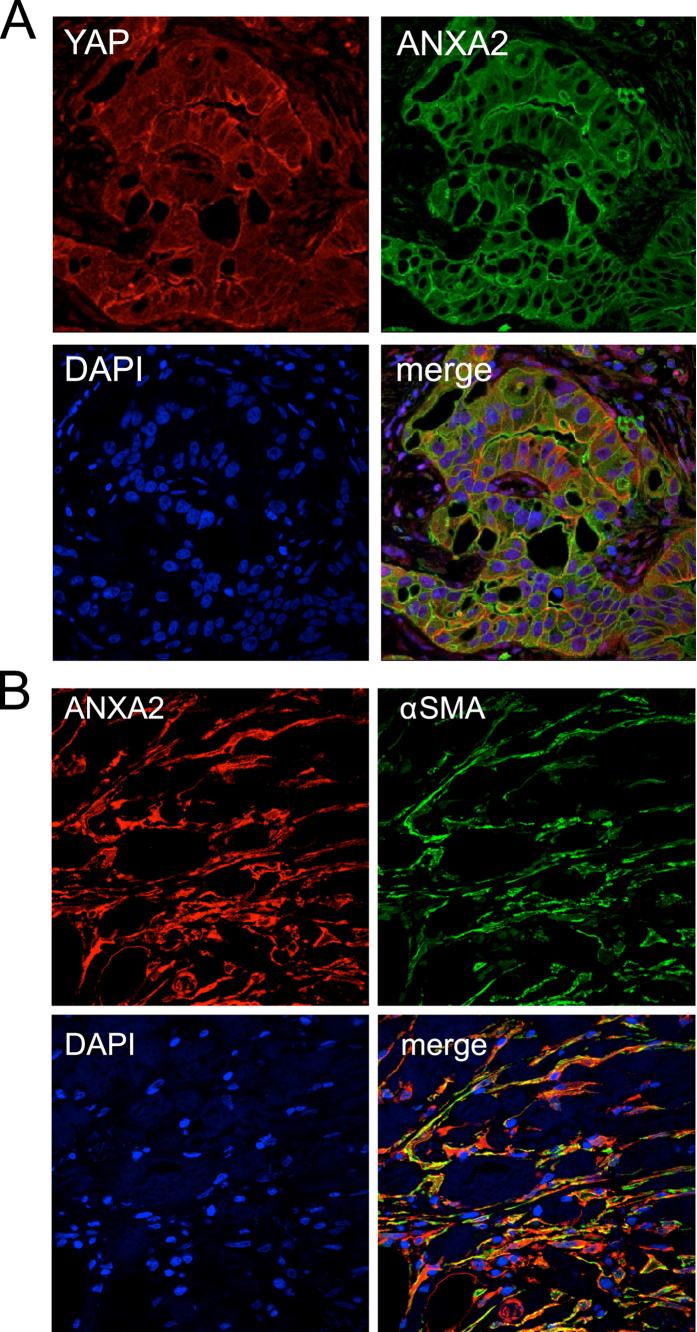
Co-expression of YAP and annexin A2 (ANXA2) in human PDAC tissues. **(A)** YAP (red), ANXA2 (green), and DAPI (blue). Note that YAP and ANXA2 are co-expressed in the cancer cells. **(B)** ANXA2 (red), α-SMA (green), and DAPI (blue). Note that the field shows predominantly stellate cells. Magnification: 63 x.

**Figure 8:**
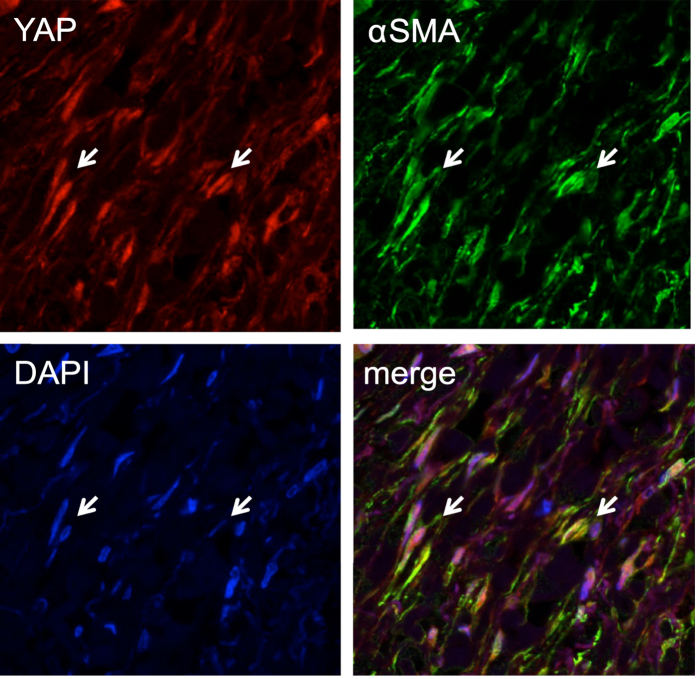
Immunofluorescence analysis of YAP in stellate cells of human chronic pancreatitis tissues. YAP (red), α-SMA (green) and DAPI (blue). The arrows indicate examples of cells in which YAP and α-SMA are co-expressed. Magnification: 63 x.

We noted that, in both chronic pancreatitis and pancreatic cancer tissues, the stromal cells stained positive for YAP and TAZ show morphology of stellate cells, which are characterized by elongated nuclei and branching cell body (Figs 3,4 and 5; Supplemental Figures 2 and 3). By immunofluorescence, we found that YAP can be detected in the same set of cells that are stained positive for α-SMA, a marker for activated stellate cells. While α-SMA is localized to the cytoplasm in these cells, YAP can be found in both the nuclei and the cytoplasm (Figs 6 and 7). Thus, our results indicate that YAP is expressed in the stellate cells of both chronic pancreatitis and PDAC. Interestingly, ANXA2 is co-expressed with YAP in activated stellate cells (Fig. 7) and in cancer cells (Fig. 8).

### YAP is up regulated in Kras G12D mutant-mediated pancreatic intraepithelial neoplastic lesion and in experimental acute and chronic pancreatitis

**Figure 9:**
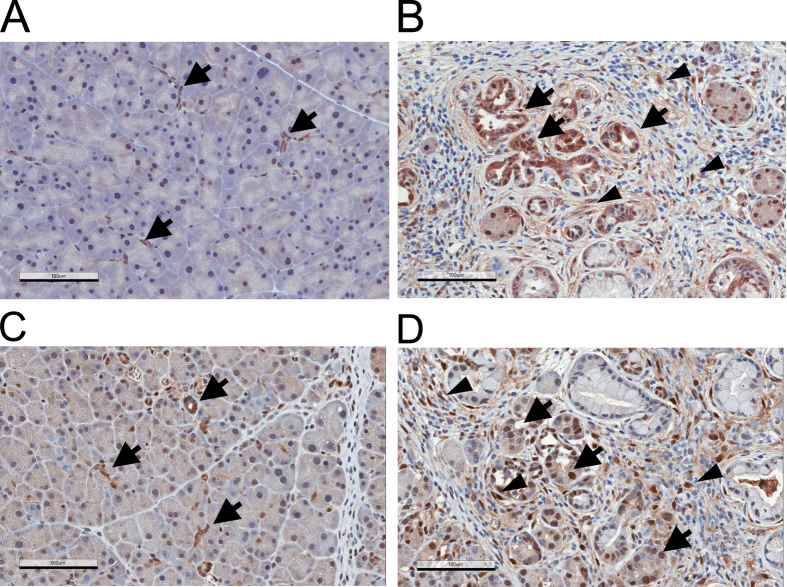
Immunohistochemistry analysis of YAP and TAZ in mouse pancreas. Immunohistochemistry analysis of mouse pancreatic tissue using anti-YAP antibody (**A,B**) or anti-TAZ (**C,D**). Representative images are shown. (**A,C**): tissues isolated from Pdx1-Cre mice; (**B,D**): tissues isolated from Pdx1-Cre/LSL-KrasG12D mice. Examples of cells with up regulation of YAP or TAZ are indicated by arrows (duct-like cells) or arrowheads (stellate cells). Magnification: 20 x, error bar: 100 μm.

The Kras gene is frequently mutated in pancreatic cancer patient and is thought to contribute to development of early neoplastic lesions^4^. In particular, the G12D mutation of Kras leads to abnormal activation of Kras functions and can promote development of PanIN in the Pdx1-Cre/LSL-KrasG12D (KC) transgenic mice, in which the KrasG12D can be induced in pancreatic cells via Pdx1 promoter-mediated expression of Cre recombinase^39^. We therefore examined YAP and TAZ expression patterns in these animals. Consistent with previous report^39^, at the age of seven month, the pancreata of KC mice show extensive areas of PanIN but no PDAC. Our IHC studies indicate that, in the control Pdx1-Cre mice, YAP and TAZ exhibit a pattern of expression in the ductal cells (Fig. 9A,C). In the KC mice, the overall expression levels of YAP and TAZ are increased and both proteins can be detected in the nuclei of PanIN cells, as well as in stellate cells associated with the neoplastic lesion (Fig. 9B,D).

**Figure 10:**
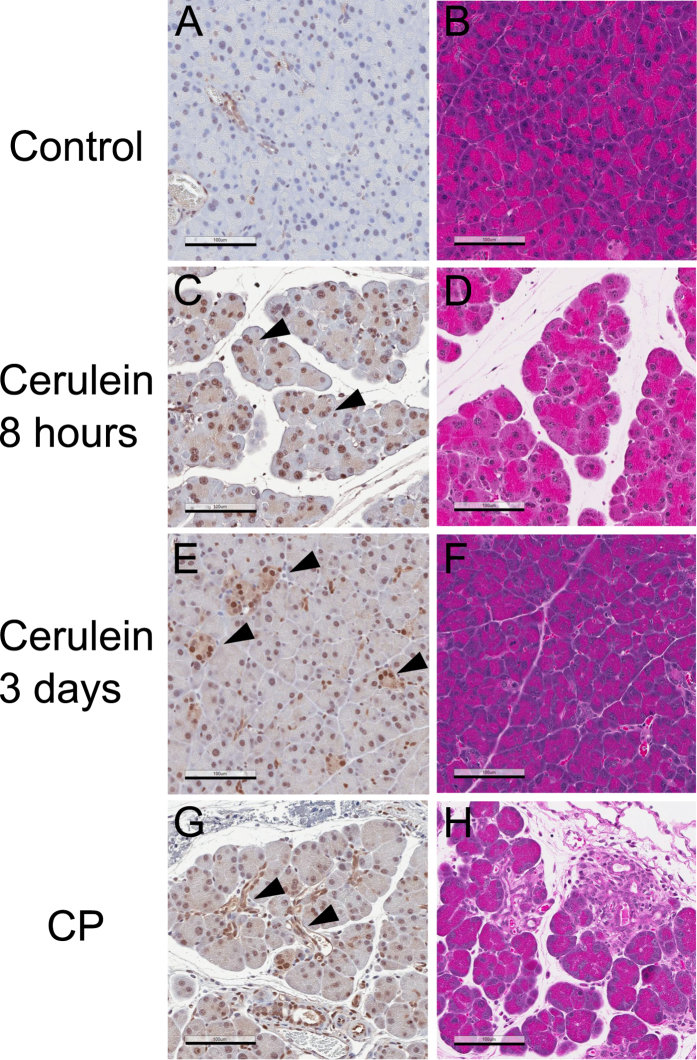
YAP is up regulated in experimental pancreatitis. Left panels: Immunohistochemistry analysis of mouse pancreatic tissues using anti-YAP antibody; Right panels: H&E staining. Representative images are shown. (**A,B**): control; (**C,D**): Eight hours after initial cerulein-induction of acute pancreatitis; (**E,F**): Three days after initial cerulein-induction of acute pancreatitis; (**G,H**): cerulein-induced chronic pancreatitis (CP). Note that H&E staining shows cerulein induced tissue damage (**D,H**) and fibrosis (**H**). The arrowheads indicate examples of cells with up regulation of YAP. Magnification: 20 x, error bar: 100 μm.

We also examined YAP levels in the process of tissue injury and regeneration using a previously established mouse model, in which acute or chronic pancreatitis develops upon exposure of mice to cerulein^6^,^40^,^41^,^42^. In particular, short-term administration of cerulein induces a mild and reversible form of acute pancreatitis in mice, characterized with edema, cellular stress, necrosis, and inflammation^6^,^40^,^42^. Our H & E staining confirmed that cerulein induced tissue injury in the pancreas by 8 hours (Fig. 10B,D). At this time point, an abrupt increase of YAP expression, with prominent nuclear localization, was detected in not only the ductal but also the acinar cells (Fig. 10C,D). YAP expression remains at high levels 3 days after the initial induction of injury, when the injured tissue has undergone extensive recovery and shows characteristics of acinar-to-ductal metaplasia (ADM) (Fig. 10E,F).

Repeated and prolonged exposure of cerulein induces a form of chronic pancreatitis^6^,^41^, which is distinguished by development of fibrosis and irreversible tissue degeneration (Fig. 10G). Under this experimental condition, YAP is up regulated, notably in the nuclei of both the acinar cells and in the expanded ductal structures that are frequently present in the chronic lesions (Fig. 10H).

## Discussion

In this study, we characterized YAP-expressing cells using cell population-specific markers (i.e. alpha-SMA for activated stellate cells, and CAII and Anxa2 for centroacinar and ductal cells) in normal and diseased pancreas. Our results indicate that YAP and TAZ expression in centroacinar and ductal cells of normal human pancreas. The centroacinar and ductal cells exhibit the capacity as multi-lineage pancreatic progenitors that are important for tissue repair and regeneration^27^,^28^,^29^,^30^,^31^,^32^. These cells express several markers previously associated with the progenitor phenotypes in embryonic pancreas and other tissues, such as Sca1, Sdf1, c-Met, nestin, and SOX9^43^,^44^,^45^. In particular, SOX-9 has been identified as a marker of pancreatic centroacinar and ductal cells and is crucial for development and regeneration^44^,^46^,^47^,^48^. The functional linkage between YAP and SOX9 has been revealed by a recent study^49^. Moreover, acinar cells exhibit regenerative potentials and undergo acinar-to-ductal metaplasia following injury of exocrine pancreas^50^,^51^,^52^. In this process, the acinar cells transdifferentiate to a precursor state that demonstrates phenotypes of ductal cells. Indeed, recent studies indicate that YAP signaling pathway is essential for development of the pancreas and activation of YAP in the adult pancreas results in pancreatitis-like phenotypes^53^,^54^. YAP and TAZ have been implicated in stem cell functions in other tissues, such as the small intestine^11^,^55^,^56^,^57^,^58^,^59^, the lung^60^,^61^, the liver^62^, the skin^63^, the bone^64^, or in developing embryos^12^,^65^. In the current study, we observed up regulation of YAP in both acinar and ductal cells, following induction of acute or chronic pancreatitis by extensive cerulein exposure. Thus, these findings suggest that YAP and TAZ may represent a set of new markers for the “stem-cell” like phenotype and play a role in modulating progenitor cell functions in pancreatic regeneration in events of pancreatic injury, such as acute or chronic pancreatitis.

We show that YAP and TAZ are expressed predominantly in the nuclei in the epithelial compartment of PDAC, which is in agreement with the notion that these proteins are functionally activated. Of note, nuclear localization patterns of YAP and TAZ expression can be readily detected in cells with morphological features of PanIN in both human tissues and Kras G12D mutant mice. These observations are consistent with the recent report that YAP plays an essential role in the early neoplastic transformation in the pancreas using a mouse model of pancreatic cancer^66^.

We previously show that YAP mediates cancer cell sensitivity to a number of therapeutics, including cisplatin, taxol, EGFR tyrosine kinase inhibitor, or small molecule inhibitor of survivin^10^. More recent studies revealed that deregulation of YAP signaling may contribute to acquired resistance to therapeutic approaches targeting the signaling events initiated by mutant Kras. For example, YAP overexpression enables pancreatic, colorectal, or lung cancer cells to overcome dependency on oncogenic Kras mutations^67^,^68^. These findings indicate that YAP may represent an attractive therapeutic target for treatment of human pancreatic cancer. It should be noted that a successful targeting strategy conceivably require simultaneous disabling YAP and WWTR1, due to their functional redundancy and the commonly overlapping expression patterns in human pancreatic cancer.

Remarkably, our results show that both YAP and TAZ are expressed in the pancreatic stellate cells associated with human pancreatic ductal adenocarcinoma and chronic pancreatitis. In particular, YAP and TAZ positive stellate cells are associated with Kras mutant-mediated early neoplastic transformation. Activation of PSCs is required to initiate fibrogenesis and chronic pancreatitis^8^. Progression to chronic pancreatitis may occur in patients with metabolic stress in the pancreas attributable to excessive alcohol consumption^69^. PSCs can be activated by growth factor or cytokines released by injured or stressed acinar cells and by various inflammatory cells^8^. For example, TGF β and CTGF/CCN2) are profibrogenic factors that can activate PSCs, resulting in their proliferation, the production and deposition of collagen 1A1 and pancreatic fibrogenesis^8^. YAP acts in concert with TGF β signaling and modulates CTGF/CCN2 expression^70^. Thus, our finding of YAP and TAZ expression in PSC of chronic pancreatitis and pancreatic cancer highlights the potential role of these transcriptional co-factors in modulating the physiological and pathological activities of PSC.

Interestingly, we found co-expression of ANXA2 with YAP/TAZ in the centroacinar and ductal cells of normal pancreas, as well as in the activated stellate cells of chronic pancreatitis and pancreatic cancer. ANXA2 is implicated in multiple functions, including the secretory pathway, tissue regeneration, and tumorigenesis. For example, ANXA2 can be localized to the cell surface and mediate the activation of plasminogen, which is involved in in thrombolysis, wound healing, angiogenesis, and EMT^71^,^72^,^73^,^74^. In addition, ANXA2 is overexpressed in pancreatic cancer and may play a critical role in cancer cell invasion and metastasis^75^,^76^,^77^. Clearly it would be of interest to define the functional interactions between ANXA2 and YAP signaling in both the normal and diseased pancreas, especially in regard to the role in the pancreatic stellate cells.

We found that YAP and TAZ are up regulated in the islets Langerhans of chronic pancreatitis tissues. Diabetes is a frequent complication in chronic pancreatitis. YAP and TAZ may be involved in regeneration or adaptive response of islets under chronic pancreatitis. A variety of islet cell types have been shown to possess the properties of progenitors, such as β-cells^78^,^79^ and nestin-positive islet-derived progenitor cells^80^. In this regard, YAP may play a role in defining the self-renewal or the regeneration capacity of the progenitor cells. Alternatively, deregulation of YAP signaling may lead to islet dysfunction. For example, a recent study showed that MST1/STK4, a mammalian orthologue of Hippo, is activated in beta cells in the islets and can induce mitochondrial-dependent apoptosis^81^. Clearly addition work is needed to clarify these possibilities.

In summary, our study demonstrates that while YAP and TAZ expression is limited to the centroacinar and intercalated ductal cells in normal human pancreas, these proteins are up regulated in PDAC and PanIN, as well as in stellate cells associated with PDAC and chronic pancreatitis. Moreover, we found expression of YAP and TAZ in the islets of Langerhans in diseased human pancreatic tissues. These findings indicate that YAP and TAZ may be involved in pancreatic tissue regeneration, and that deregulation of these proteins may play a role in neoplastic transformation and stellate cell functions in both PDAC and pancreatitis.

## Methods

### Antibodies

The antibodies used in this study include: anti-YAP and anti-phospho S127 YAP (#4912; #4911; and #13008, Cell Signaling Technology, Danvers, MA) anti-YAP (H-125), anti-ANXA2 (C-10), and anti-CA II (Santa Cruz Biotechnology, Santa Cruz, CA); anti-WWTR1/TAZ and anti α-SMA (Sigma, St. Louis, MO); anti-MST1R/RON antibodies (R&D Systems, Minneapolis, MN); horse radish peroxidase-conjugated secondary antibodies against mouse or rabbit were from Dako (Carpentaria, CA). Alexa Fluor 488 or 594 conjugated anti-mouse or anti-rabbit antibodies were purchased from Life Technologies (Grand Island, NY).

### Human Tissues

Formalin-fixed and paraffin-embedded (FFPE) human pancreatic specimens were obtained from the Cedars-Sinai Pathology archive and biorepository, and the analyses were carried out under protocols approved by the Internal Review Board at the Cedars-Sinai Medical Center (IRB protocols #4201, #28197 and #34086). All experiments were performed in accordance with relevant guidelines and regulations.

### Transgenic Mice and Experimental Acute and Chronic Pancreatitis

The Pdx1-Cre and Lox-Stop-Lox-KrasG12D mice were described previously^39^. The mice were interbred to create Pdx1-CRE/LSL-KrasG12D (KC) mice at the Genetics Core of the Cedars-Sinai Medical Center. Pancreata were collected from 7-month old mice, fixed in formalin and embedded in paraffin.

C57BL/6 J mice (B6J) were obtained from the Jackson Laboratory (Bar Harbor, ME). To induce acute pancreatitis, the mice were given seven hourly intraperitoneal (IP) injections of cerulein (American Peptide Company, Sunnyvale, CA), at a dosage of 50 μg/kg body weight per injection. The mice subjected to injections of PBS served as the control. The mice were sacrificed at the time points as indicated in each experiment (ranging from 8 hours to 3 days after the initial injection), and the tissues were collected used for further analysis. To induce acute pancreatitis, the mice received two courses of cerulein injection per week (with 3–4 days apart) for a total of 5 weeks. Each course of cerulein administration consisted of seven hourly intraperitoneal injections of cerulein at a dosage of 50 μg/kg body weight per injection. A separate set of mice will receive sterile saline solution as control. The mice were sacrificed five days after the final injection, and the tissues were collected used for further analysis. Pancreata were fixed in formalin and embedded in paraffin.

All animal procedures were approved by the Cedars-Sinai Institutional Animal Care and Use Committee (IACUC) under protocol number 3935. All experiments were performed in accordance with relevant guidelines and regulations.

### Immunohistochemistry

For the study of human tissues, a total of 4 sets of specimens, each contain normal, PDAC, and chronic pancreatitis tissues, were analyzed. Both human and mouse FFPE pancreatic specimens were de-paraffinized, rehydrated, and subjected to antigen retrieval by heating. After incubating in blocking solution from VECTASTAIN Elite ABC Kit (Vector Laboratories, Burlingame, CA) for 20 minutes, the samples were treated with the primary antibodies for 60 minutes or overnight. The sections were then washed three times in PBS, followed by incubation with a secondary antibody conjugated with horse radish peroxidase (Dako, Carpentaria, CA) for 60 minutes. The samples were subjected to final washes, and specific stains were developed with ABC reagent for up to 10 minutes, using ImmPACT/DAB peroxidase substrate (Vector Laboratories, Burlingame, CA). The slides were mounted and scanned using Aperio Scanscope**®** AT Turbo (Leica Microsystems, Buffalo Grove, IL).

### Immunofluorescence and confocal microscopy

The FFPE pancreatic tissue sections prepared on glass slides were de-paraffinized, rehydrated, and subjected to antigen retrieval by heating. After incubating in blocking solution (Animal-Free Blocker™, Vector Laboratories), the sections were stained with the primary and secondary antibodies sequentially in phosphate-buffered saline (PBS) containing 0.2% Triton X-100. The slides were mounted and analyzed using Leica TCS SP5 confocal microscope (Leica Microsystems, Buffalo Grove, IL). For analysis of gene co-expression in the IF study, GraphPAD Prism software (GraphPad Software, San Diego, CA, USA) was used to perform Fisher’s exact test using data collected in 3 random fields of each immunofluorescence staining sample.

## Additional Information

**How to cite this article**: Morvaridi, S. *et al.* Role of YAP and TAZ in pancreatic ductal adenocarcinoma and in stellate cells associated with cancer and chronic pancreatitis. *Sci. Rep.* **5**, 16759; doi: 10.1038/srep16759 (2015).

## Supplementary Material

Supplementary Information
