# Supplementary material for: Role of YAP and TAZ in pancreatic ductal adenocarcinoma and in stellate cells associated with cancer and chronic pancreatitis
Source: Sci Rep. 2015 Nov 16;5:16759. doi: 10.1038/srep16759 (PMC4645184; doi:10.1038/srep16759)

Supplemental information

Role of YAP and TAZ in pancreatic ductal adenocarcinoma and in stellate cells associated with cancer and chronic pancreatitis

Susan Morvaridi[1, 2], Deepti Dhall[3], Mark I. Greene[4], Stephen Pandol[1, 2] and Qiang Wang[1, 2, *]

[1]Department of Medicine; [2]Pancreatic Research Program; [3]Department of Pathology and Laboratory Medicine; Cedars-Sinai Medical Center, Los Angeles, CA 90048;

[4]Department of Pathology and Laboratory Medicine, Perelman School of Medicine, University of Pennsylvania, Philadelphia, PA 19104

*Corresponding author:

Qiang Wang, Ph.D.

Department of Medicine, Pancreatic Research Program, Cedars-Sinai Medical Center, 8700 Beverly Boulevard, Los Angeles, CA, USA 90048.

Phone: 310-423-7638, Fax: 310-248-6799,

Email: qiang.wang@cshs.org

Supplemental Figure Legends

Figure S1. Immunohistochemistry analysis of YAP in normal human pancreas using three additional anti-YAP antibodies (A) anti-YAP polyclonal Ab (Cell Signaling Technology #4912). **(B)** Anti-phospho S127 YAP polyclonal Ab (Cell Signaling Technology #4911); **(C)** Anti-phospho S127 YAP monoclonal Rabbit Ab (Cell Signaling Technology #13008; D9W2I). Magnification: 20x, error bar: 100 µm.

Figure S2. IHC analysis of YAP expression in human PDAC tissues (anti-YAP antibody, H125). Representative images of specimens obtained from three individuals are shown. Magnification: 20x, error bar: 100 µm.

Figure S3. YAP expression in human chronic pancreatitis tissues (anti-YAP antibody, H125). Representative images of specimens obtained from three individuals are shown. Magnification: 20x, error bar: 100 µm.

Figure S4. IHC staining of ANXA2 expression in human normal, chronic pancreatitis, and PDAC tissues. Representative images are shown. Magnification: 20x, error bar: 100 µm.

Supplemental Table S1

Association of CA II and ANXA2 expression in normal pancreas.

	CA II positive	CA II negative	P value
ANXA2 positive	345	24	<0.0001
ANXA2 negative	45	618	

Association of YAP and ANXA2 expression in normal pancreas

	YAP positive	YAP negative	P value
ANXA2 positive	315	12	<0.0001
ANXA2 negative	30	582	

Three random fields of immunofluorescence staining were examined. Fisher's exact test was performed using GraphPAD Prism software. The total number of cells in each category and the P values are shown.

Supplemental Fig. 1   Morvaridi et al.

A

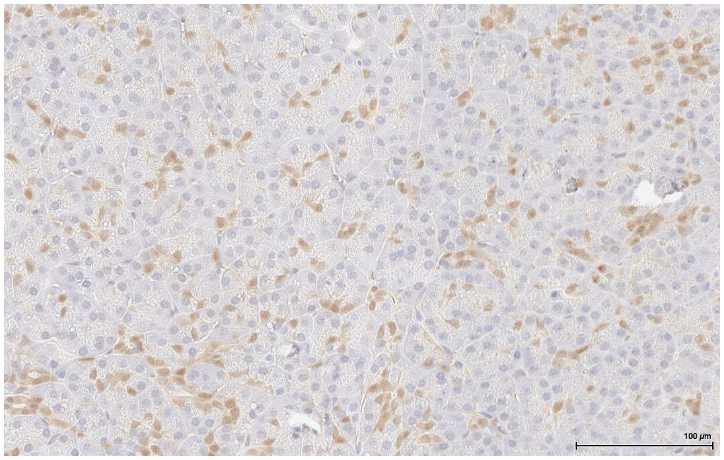

B

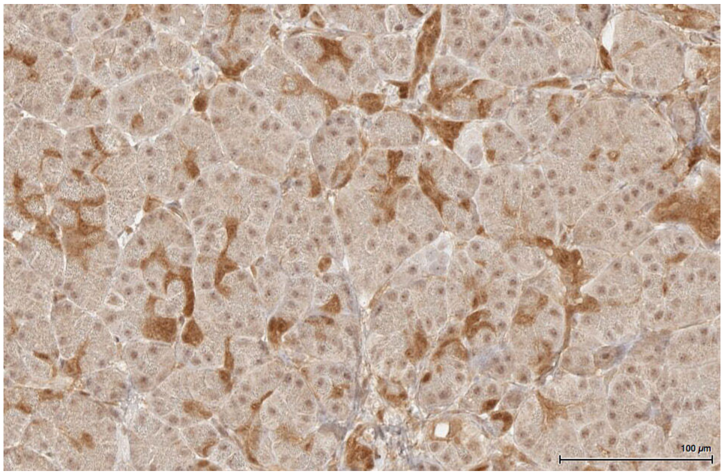

C

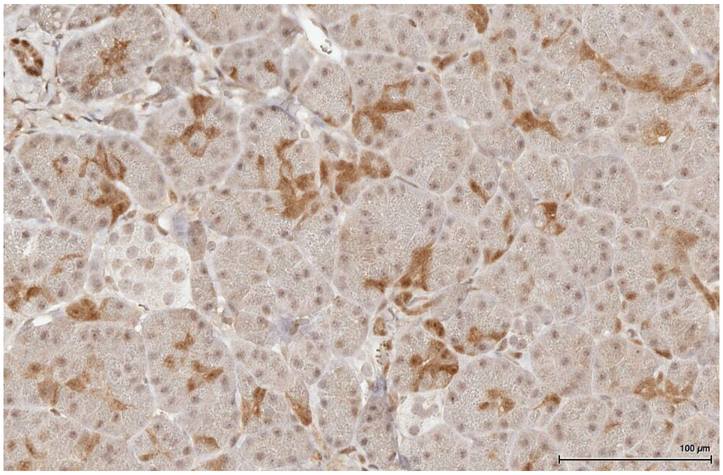

Supplemental Fig. 2 Morvaridi et al.

PDAC #2

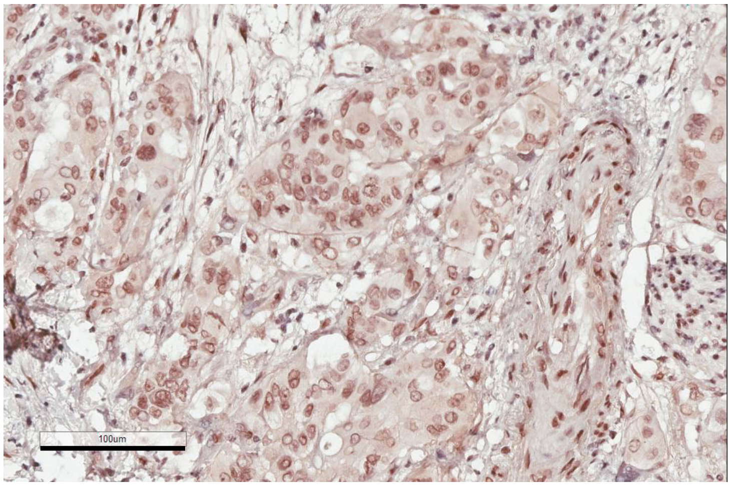

PDAC #3

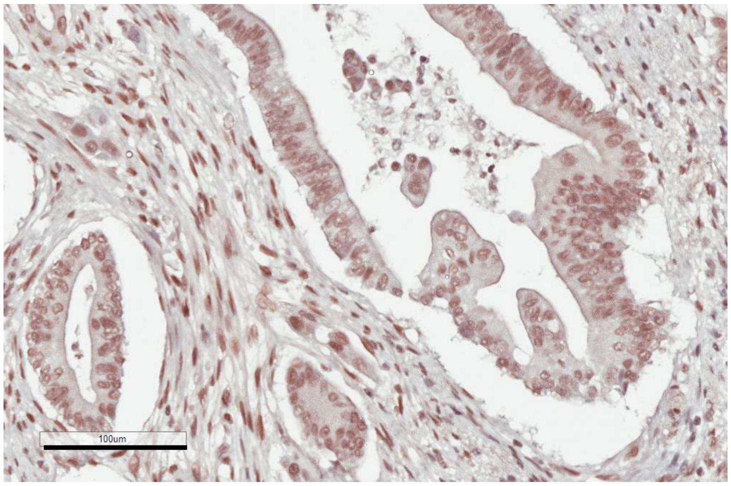

PDAC #4

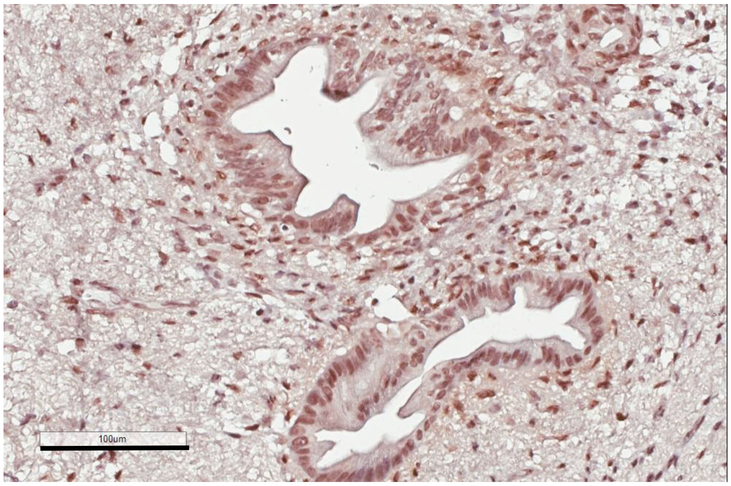

Supplemental Fig. 3 Morvaridi et al.

CP #2

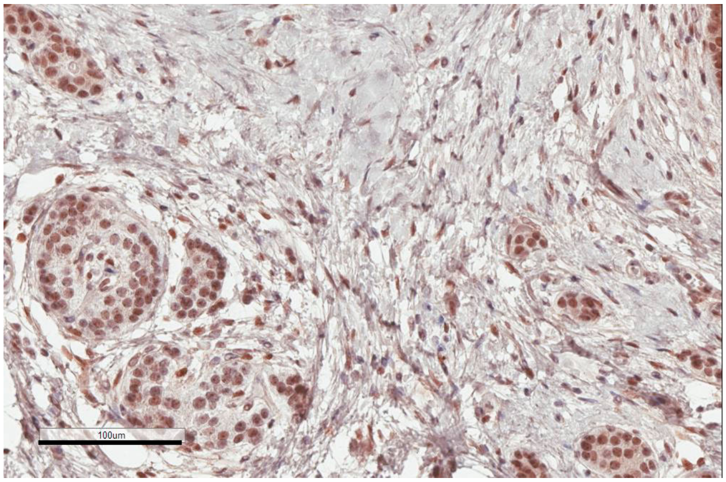

CP #3

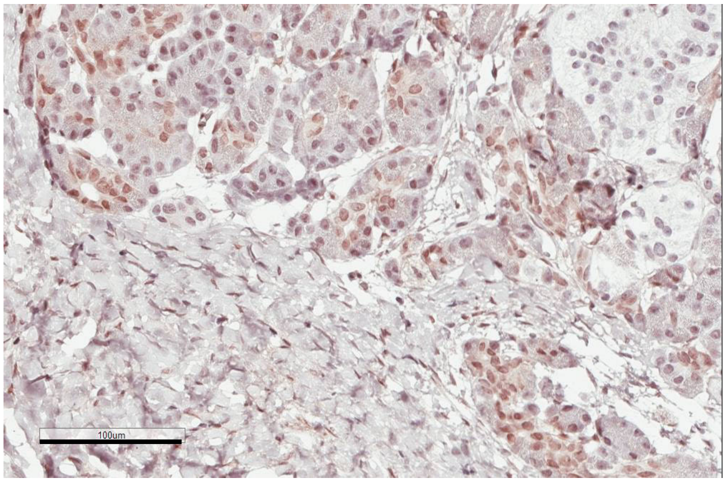

CP #4

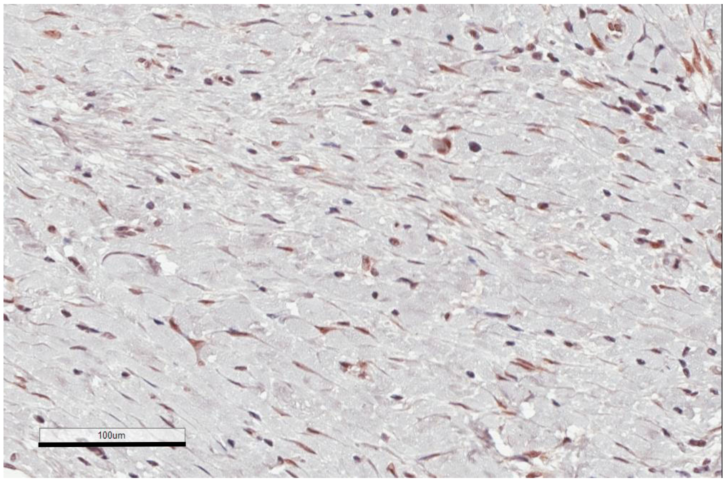

Supplemental Fig. 4 Morvaridi et al.

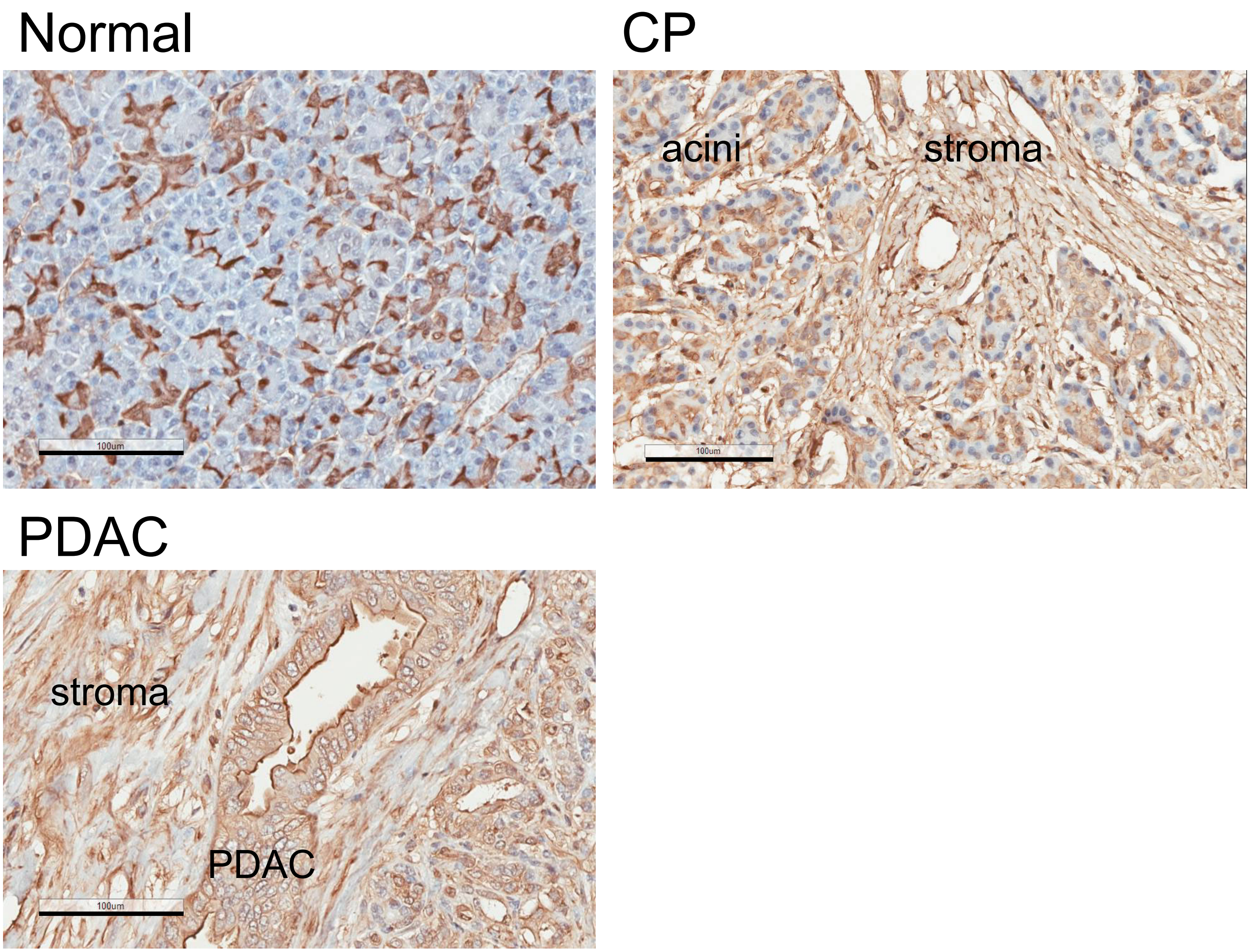

Supplement: Supplementary Information [file srep16759-s1.pdf]
